# Chronic Nephritis without Albuminuria

**Published:** 1903-10-10

**Authors:** 


### CHRONIC NEPHRITIS WITHOUT ALBUMINURIA.

While the fact that albumin may be temporarily absent from the urine during chronic interstitial nephritis is well known, it is perhaps not so fully appreciated that nephritis may progress to an immediately dangerous stage, with continued absence of albuminuria. Dr. Arthur R. Elliott[1] reports some cases which illustrate this point. In testing the urine he used three methods in every case so as to lessen the chance of error; the reagents used were potassium-ferrocyanide, cold nitric acid, and heat with acetic acid. He purposely avoided the extremely delicate tests, such as the potassio-mercuric iodide one, because he considers them to be too sensitive for clinical purposes. The first case was that of a man thirty years of age, who first consulted a medical practitioner because of visual disturbance. He was found to have neuro-retinitis of albuminuric type. The only other symptoms of ill-health which the patient complained of were some dyspnœa and slight precordial uneasiness upon exertion. Physical examination revealed moderate but distinct cardiac hypertrophy, and marked arterial fibrosis with increased tension. The urine was entirely free from morbid characteristics, except for the presence of hyaline casts and a reduction of urinary solids below the average. During the subsequent 28 months the urine never showed the least trace of albumin although a large number of 24-hour specimens were examined. In another case occurring in a woman 52 years of age, the clinical history of the patient's nephritis commenced with the sudden development of uræmic convulsions, from which she was with difficulty rescued. She lived for 3½ years, and then succumbed to a second attack of uræmia. During the interval frequent examinations of the urine failed to detect albumin. Hyaline casts were constantly to be found, although they were not present in large numbers. Cardio-vascular changes were well developed, and there was marked polyuria, the quantity passed in 24 hours often being as much as 100 ounces. The total urea and other urinary solids was reduced. Dr. Elliott gives notes of three other cases of chronic nephritis in which there was absence of albuminuria. Commenting on his cases he lays great importance on the diagnostic significance of casts, and thinks it is not too much to say that chronic interstitial nephritis never exists as a clinically recognisable condition without the presence of casts in the urine. The casts are most frequently hyaline, and they must be looked for with care as they are usually present only in small numbers. Cardio-vascular changes are also of the greatest value, from a diagnostic point of view, for they are comparatively easy to observe, while however early a definite case of chronic interstitial nephritis may come under observation the pulse tension is high and the artery thickened. Again, if it be found that a patient has developed the habit of getting out of bed to empty his bladder, and that the necessity for doing this has arisen apart from any obvious cause, such as the taking of excess of fluids shortly before retiring, the symptom is highly suggestive of chronic interstitial nephritis. The presence of albumin in the urine in this disease is uncertain, and it is therefore a very unreliable diagnostic guide. When present with the other symptoms it serves to make the diagnosis more certain, but if absent no contrary inference is justifiable, and the diagnosis must be made without its aid. Much more important than the presence of albumin is a lowered excretion of urea, which may exist for months or years before any albuminuria occurs.

[1] Medical News, Sept. 19.

